# Tracking Sensory Characteristics of Virgin Olive Oils During Storage: Interpretation of Their Changes from a Multiparametric Perspective

**DOI:** 10.3390/molecules25071686

**Published:** 2020-04-07

**Authors:** Ana Lobo-Prieto, Noelia Tena, Ramón Aparicio-Ruiz, María T. Morales, Diego L. García-González

**Affiliations:** 1 Instituto de la Grasa (CSIC), Ctra. de Utrera, km. 1, Campus Universitario Pablo de Olavide-building 46, 41013 Sevilla, Spain; ana.lobo@ig.csic.es; 2 Department of Analytical Chemistry, Faculty of Pharmacy, University of Seville, Prof. García González, 2, 41012 Seville, Spain; noelia.tena@ig.csic.es (N.T.); aparicioruiz@cica.es (R.A.-R.); tmorales@us.es (M.T.M.)

**Keywords:** virgin olive oil, volatile compounds, sensory assessment, storage, SPME-GC, oxidation, oxidative stability, mesh cell-FTIR

## Abstract

Virgin olive oil is inevitably subject to an oxidation process during storage that can affect its stability and quality due to off-flavors that develop before the oil surpasses its ‘best before’ date. Many parameters are involved in the oxidation process at moderate conditions. Therefore, a multiparametric study is necessary to establish a link between physico-chemical changes and sensory quality degradation in a real storage experiment. In this context, a storage experiment of 27 months was performed for four monovarietal virgin olive oils, bottled in transparent 500-mL PET bottles and subjected to conditions close to a supermarket scenario. Volatile composition, quality parameters and phenolic compounds were determined monthly. Simultaneously, an accredited sensory panel assessed their sensory characteristics. The stability of the fresh samples was also studied with the oxidative stability index (OSI) and mesh cell-FTIR. (*E*)-2-hexenal, (*Z*)-3-hexen-1-ol and (*E*)-2-hexen-1-ol were identified as markers of the fruity attribute. Hexanal and nonanal were also identified as compounds that were associated with the rise of median of defect during storage. Some disagreements were observed between the sensory assessment and the OSI analyzed by Rancimat. However, the increase of concentration of rancid markers agreed with the increase of aldehyde band measured with mesh cell-FTIR.

## 1. Introduction

The production of virgin olive oil (VOO) is limited to several months per year; this leads to the necessity of storing the oil to ensure a continuous supply for consumers. This storage is carried out at various levels in the food chain, for example, in tanks during trading, or in bottles in retailers. During storage, VOO is exposed to external variables that cause changes to its composition that lead to a loss of its nutritional quality and changes of its sensory characteristics [1]. It is well known that VOO is more resistant to oxidation than other edible oils because of its composition. In spite of this, numerous studies [2,3,4,5] have demonstrated that storage conditions have a strong influence in the degradations of oils that may cause some problems in retailing. Thus, light and temperature—even in mild conditions—can affect considerably the stability and quality of VOO [6,7,8,9,10,11] during the shelf life, leading to a loss of its nutritional properties and ultimately, a development of off-flavors that result in rancidity. This problem of stability has sometimes led to discrepancies between the results of control-testing and information declared on the label. This is the case of some extra virgin olive oils that—after a storage period—may be unexpectedly downgraded to virgin olive oil category. For this reason, regulatory bodies have stablished some specific requirements concerning the storage conditions of olive oil [12,13]. Additionally, International Olive Council has recently approved a document with the best practice guidelines for the storage of VOO [14].

The need of a major control of VOO stability is not new, although the increasing importance of sensory quality of this product and the stricter standards today [15] have encouraged producers and standardization bodies to find new methods to evaluate and understand VOO stability. Some methods are based on the study of the quality parameters during oil storage under conditions that are similar to real ones. These methods require a long period of study (several months), so they are not useful when a rapid answer is required. Other methods use accelerated conditions to obtain results in a shorter time (i.e., several hours/days), such as Rancimat, but they accelerate the oxidation process by means of drastic conditions and the results do not correlate well with the real oxidation process and do not take photooxidation into account [7].

Several studies have proposed rapid methods that assess VOO stability under conditions that are close to the real ones. Thus, Schaal oven test allows determining stability of the oils at 63 °C [16]. Other techniques, such as electron spin resonance spectroscopy [17,18] and differential scanning calorimetry [19], have been also used to assess oil stability at 70 °C or lower temperatures. Recently, our group developed a new procedure based on mesh cell-FTIR spectroscopy [6] to assess VOO stability at room temperature considering the effect of photooxidation [7,20]. The methods that use moderate conditions provide information that is easily correlated with the real degradation taking place in VOO under real storage. However, the interpretation of results is hindered by the fact that many parameters (chemical, physico-chemical, sensory) are evolving at the same time with different kinetics. Thus, it is difficult to establish a simple rule based on these parameters to assess if an oil is clearly out of the ‘best before’ date for its consumption. A comprehensive study of all these parameters and their inter-relationships among them is necessary to understand VOO shelf-life.

In the last decade, numerous authors have focused on tracking different compounds and parameters of VOO during its shelf life. The majority of these studies have aimed to monitor the changes in the quality parameters of VOO during the storage under different conditions and with different types of containers [5,21]. Other authors have centered their studies on the loss of VOO healthy compounds, such as phenols, during the shelf life under different storage conditions [22,23,24]. Due to the changes in sensory characteristics that take place during shelf-life, some of the studies were based on volatile analysis during storage at different conditions [3,8,25,26]. Some of these studies do not use conditions that are commonly used in a supermarket, where oils are exposed to room temperature and light/dark cycles of 12 h. Therefore, currently a disparity of results is observed when the volatile compounds and other parameters are evaluated during the storage of VOOs, with a resulting difficulty in the interpretation of oxidation processes at moderate conditions.

Aroma has a strong influence on the consumer’s rejection or acceptability of VOOs that have been stored for several months [7]. For this reason, stablishing a link between the chemical changes taking place in the oils during the storage and the sensory changes during this time is needed to predict better the ‘best before’ date of the oil. The aim of this study was to evaluate the VOO quality changes generated by the oxidation process to which the oil is inevitably subjected during its storage. Thus, a long storage experiment of 27 months was performed, period during which VOOs from three different cultivars were exposed under moderate conditions simulating a supermarket scenario. When moderate conditions of temperature and light intensity are used, the changes on sensory properties can be subtle and difficult to interpret. In order to facilitate this interpretation, in addition to the volatile composition and sensory assessment, chemical parameters directly or indirectly related to virgin olive oil quality were analyzed month by month. Furthermore, the oxidative stabilities of fresh oils evaluated with accelerated procedures (Rancimat and mesh cell-FTIR) were also considered. With all this information, the moments in which a remarkable sensory change take place was identified and explained by changes produced in the volatile composition and the other indexes of quality. Finally, the results of stability assessment on the fresh samples carried out by Rancimat and mesh-cell FTIR were analyzed according to the development of the off-flavors during the storage of the four monocultivar samples.

## 2. Results and Discussion

### 2.1. Characterization of Fresh VOOs

Four VOOs were selected from three different cultivars (Hojiblanca, Picual and two from Arbequina) for this study. These cultivars were selected to cover different chemical composition and for their genuine sensory characteristics [27,28].

The quality parameters were determined for the fresh four VOOs before starting the storage (“time zero”) with the aim of characterizing their actual quality at the moment of bottling. Free acidity, extinction coefficients (K_270_ and K_232_), peroxide value (PV), fatty acid composition, total phenol content, and results from Rancimat and sensory assessment (medians of the defect and the fruity attribute) are shown in Table 1, together with date of extraction and cultivar of each VOO. According to the results obtained for PV, K_270_ and K_232_, all the values were below the limits stated in EC regulation [13] for the classification as “extra virgin olive oil” category. However, the sample VOO2 was pointed out as the most oxidized sample before starting the experiment despite all the samples were collected from the vertical centrifuge and the storage experiment started shortly after. Thus, in all these parameters, VOO2 showed the highest values, although far from the maximum limits for the “extra virgin olive oil” category. However, the sensory quality parameters (medians of defect and the fruity attribute showed in Table 1) revealed that VOO4 was initially categorized within the “virgin olive oil” category instead of “extra virgin olive oil” category. In this sample, panelists detected a winey-vinegary defect (median of defect = 2.1) before starting the storage. The organoleptic assessment before the storage experiment reported the sensory differences associated to the cultivars [27,28]. Thus, assessors identified an intense fruity and green odor in the fresh sample of VOO1 (VOO1-0m), which explained the highest median value for the fruity attribute (Table 1). VOO3-0m was characterized by a high median of the fruity attribute and the panelists described it as a fruity, bitter and pungent oil with some fig and wood notes, typical from Picual cultivar [29]. Whereas, VOO2-0m and VOO4-0m, from Arbequina cultivar, showed a delicate fruitiness with slight bitter and pungent notes, showing the lowest median of the fruity attribute.

In order to assess the oil susceptibility to oxidation, the content of total phenols and the fatty acids composition were determined (Table 1). Furthermore, the oil stability index (OSI) was determined by Rancimat method, which values are shown in Table 1. The results show the following stability order of the oils (from more to less stable): VOO3, VOO4, VOO1, VOO2. Thus, VOO3 showed the highest oxidative stability (82.80 h), which can be explained by its high concentration of phenols (534.82 mg/kg) and monounsaturated fatty acids (81.82%). VOO2 and VOO4, which were characterized by a medium phenol content and the highest percentage of polyunsaturated fatty acids (Table 1), showed totally different oxidative stability values between them. Thus, VOO4 showed better stability (53.60 h) than VOO2 (22.95 h), the former showing a higher phenol concentration (468.10 mg/kg) than the latter (338.90 mg/kg). Sample VOO1 showed an intermediate situation because its oxidative stability value was 38.71 h. Although this sample had a high monounsaturated fatty acid percentage, it showed the lowest total phenol content (226.71 mg/kg).

Regarding the volatile composition, Table 2 shows the concentration of the identified volatile compounds of the fresh oils before starting the storage experiment, their odor thresholds and their sensory attributes. These volatile concentrations provide useful information about their oxidation state or the presence of some oxidative/fermentative defects [1,3,30,31] before the storage. Table 2 shows the high content of C6 aliphatic compounds, such as hexanal, (*E*)-2-hexenal, hexyl acetate, hexanol, (*E*)-3-hexen-1-ol, (*Z*)-3-hexen-1-ol, (*E*)-2-hexen-1-ol, (*Z*)-2-hexen-1-ol and (*Z*)-3-hexenyl acetate, which derived from linoleic and linolenic acids through the lipoxygenase (LOX) pathway [1,32,33]. The total concentration for this group of compounds, which provide pleasant notes to the oil, represented more than 20% of the total concentration of volatiles in all samples. Thus, the highest concentration for C6 lipoxygenase products was found in VOO1-0m, representing 33% of its total volatile compounds, with a value of 18.26 mg/kg. Whereas, the percentages and concentrations values for the rest of samples were 25.33% and 11.13 mg/kg for VOO2-0m, 24.41% and 15.45 mg/kg for VOO3-0m and 25.90% and 11.09 mg/kg for VOO4-0m. (*E*)-2-hexanal is one of the most abundant compounds in all fresh samples, with a concentration value that ranged from 4.53 to 5.81 mg/kg. Hexanal and hexanol showed high concentration values as well, in the range of 1.08–3.83 mg/kg. These three compounds strongly contributed to the aroma of all fresh samples since their concentrations exceeded their odor threshold value (Table 2). The high amount of (*Z*)-3-hexen-1-ol in VOO1 (1.10 mg/kg) and (*Z*)-3-hexenyl acetate in VOO3 (1.73 mg/kg) are also remarkable. These compounds are characterized by ripe fruity, bitter and green sensory attributes.

On the other hand, the analysis of the fresh samples pointed out that the compounds responsible for rancid defect [31], such as heptanal, octanal, (*E*)-2-heptanal, nonanal and (*E*)-2-decenal were found at low concentrations before the storage. The Hojiblanca oil (VOO1-0m) was characterized by the lowest concentration of octanal (0.07 mg/kg for VOO1-0m vs. 0.42 mg/kg, 1.01 mg/kg and 0.55 mg/kg for VOO2-0m, VOO3-0m and VOO4-0m, respectively). The total amount of carboxylic acids also pointed out a higher degradation of VOO2-0m (12.02 mg/kg) and VOO3-0m (15.75 mg/kg) oils compared to VOO1-0m (6.41 mg/kg) and VOO4-0m (3.97 mg/kg). Furthermore, ethanol and acetic acid, which are typically found at high concentrations in oils with fermentative defects (e.g., winey-vinegary defect) [31,34], were identified in the fresh samples, although their concentrations were not enough to produce a remarkable sensory impact. Thus, the concentrations of ethanol were lower than its odor threshold in all cases. On the contrary, in the case of acetic acid, the concentrations were higher than the odor threshold in all the oils (Table 2). Only VOO2-0m showed a particularly high concentration of acetic acid (5.16 mg/kg), which was at least 2 times the concentration found in the other oils (Table 2).

### 2.2. Chemical Changes during the Storage Experiment

In order to assess the chemical changes that take place during the VOO shelf life, a storage experiment was carried out for 27 months under controlled conditions (see Section 3.1). Quality parameters, volatile composition and sensory characteristics (panel test) were monthly determined during this period of storage.

**Figure 1:**
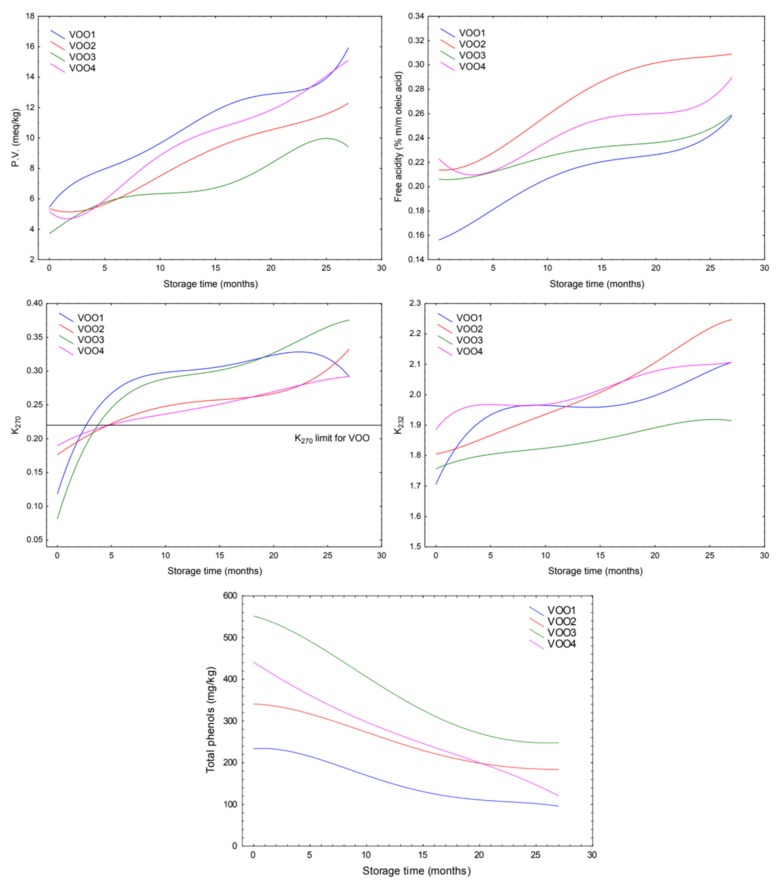
Time-course plots of peroxide value (PV), free acidity (FFA), ultraviolet absorbance at 270 nm (K_270_) and 232 nm (K_232_) and total phenols concentration per each virgin olive oil (VOO) (polynomial fitting). According to [13]: Limits for extra virgin olive oil (EVOO): PV ≤ 20 meq O_2_/kg, FFA ≤ 0.8%, K_270_ ≤ 0.22, K_232_ ≤ 2.50. Limits for virgin olive oil (VOO): PV ≤ 20 meq O_2_/kg, FFA ≤ 2.0%, K_270_ ≤ 0.25, K_232_ ≤ 2.60.

The indicators of the VOO quality alteration showed an increase during the storage experiment at moderate conditions. Figure 1 shows the evolution of quality parameters and total phenol concentration per each sample during the long-term storage.

In all cases, the final values were inside the “extra virgin olive oil” category according to the limits stated in EC regulation [13], except for K_270_. Thus, this parameter exceeded the legislative limit stablished for the “extra virgin olive oil” category in the first months of storage (1–5 month) for the four VOOs. Furthermore, the increase of the K_270_ is faster in VOO1 and VOO3 than in the other two VOOs in the first five months of storage. On the other hand, the total phenol concentration decreased during the storage experiment in all cases. Their concentrations showed a higher decrease during the first twenty months of storage compared to the seven last months in all VOO. These results agree with the results found by other authors [22,23]. VOO3 and VOO4 underwent the highest concentration decrease, which were respectively 297.21 and 329.88 mg/kg. The other two samples showed a slighter decrease of their concentrations, with a reduction value of 133.24 mg/kg for VOO1 and 155.09 mg/kg for VOO2.

**Figure 2:**
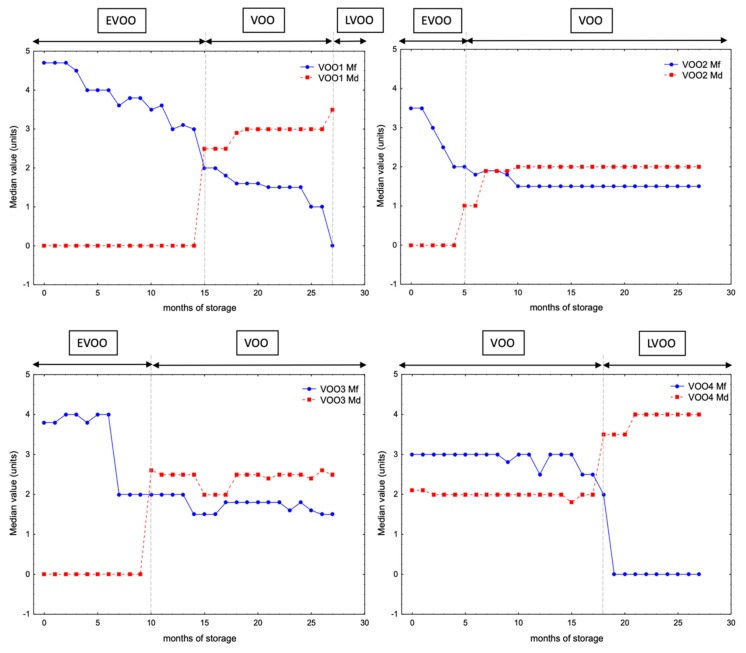
Changes of the oil category, the median of the fruity attribute (Mf) and the median of defect (Md) for the four virgin olive oils (VOO1–VOO4) during the storage.

In the course of the storage experiment, panelists identified some flavor changes in the samples, which resulted in a variation in their medians of the fruity attribute and the defect. Figure 2 shows the evolution of the medians of the defect and the fruity attributes for each sample during the storage. This figure also shows the variations of VOO category of the oils during the storage, according to the limits established in European regulation [13]. The sensory assessment results revealed that the pleasant odor attributes decreased during the storage. In all cases, a reduction of the median of the fruity attributes was observed. VOO2 showed the fastest decrease, so displaying a drop from 3.5 to 2.0 units in the median of the fruity attribute during the first five months of storage, while the rest of the samples kept their initial values at this time. On the other hand, the median of defect showed that VOO2 was downgraded to “virgin olive oil” category rapidly, in the fifth month of storage (VOO2-5m) because assessors detected a winey-vinegary defect at this time. The time-trend changes of K_232_ and FFA also pointed out that this sample was the least stable, since the final values of these parameters (2.45% and 0.32% respectively) were the highest compared with the rest of the VOOs (Figure 1). The next VOO undergoing a downgrade of category was VOO3. Thus, this oil changed to “virgin olive oil” category in the tenth month of storage (VOO3-10m) when assessors detected a winey-vinegary defect as well. Although the evolution of K_270_ for this oil pointed out that this oil was unstable (the final value after 27 months of storage was 0.38), the time-trend changes of the other parameters pointed out its stability during the storage. Thus, this oil showed the lowest values of K_232_, PV and FFA compared with the rest of the stored VOOs at the end of the storage period (Figure 1). The off-flavors detected by the panelist after 10 months may have been in the oil from the beginning of the storage and been masked by the high intensity of green and fruity attributes (Figure 2). Finally, VOO1 changed to “virgin olive oil” category in the fifteenth month of storage (VOO1-15m), because an incipient rancid defect was detected, this sample being the one that needed more time to undergo a change of category. These results do not match with the low phenol content of this VOO and its rapid evolution of PV and K_270_ (Figure 1). Furthermore, despite the changes in the medians of the fruity attribute and the defect were more moderate during the last months (months 15–27) than in the first ones (months 0–14) in VOO1 (Figure 2), this sample underwent another change of category to “lampante virgin olive oil” at month 27 (VOO1-27m). This oil was identified as the least stable according to the evolution of PV, which is associated with the first step of the oxidation process and reached its maximum value (15.27 meq O_2_/kg) at the end of the storage period. With respect to VOO4, which was initially categorized as “virgin olive oil”, this oil showed an incipient rancidity in the fourth month of storage and the median of defect raised above 3.5 in month 18 (VOO4-18m) and consequently the oil was downgraded to “lampante virgin olive oil” category at this time.

The total concentration of volatile compounds is showed in Table 2, where the initial (month 0) and final values (month 27) per sample are displayed. Furthermore, Tables S1–S4 (Supplementary Material) show the concentrations of the volatile compounds in the VOOs during the storage experiment. These values revealed a moderate change in the total concentration of volatiles during the storage time. Thus, a maximum of 15% of variation was observed when comparing the total concentration of volatile compounds between the beginning and the end of the storage experiment (Table 2). However, the panelists detected important changes in the sensory characteristics of the samples that led to a change in the category. In order to extract more information about the changes taking place during the storage, the total concentration of volatiles was studied regarding 5 different chemical series: aldehydes, alcohols, esters, ketones and carboxylic acids. Table S5 shows the concentration of these chemical series at the beginning and the end of the storage. In this case, the maximum variation of concentration was found in carboxylic acids. Thus, the concentration of this chemical series was up to 54.56% higher at the end of the storage experiment in VOO4 (Table S5). The maximum percentages of variation for the other chemical series were 39.40% for aldehydes (VOO1), 43.18% for alcohols (VOO2), 37.26% for esters (VOO4) and 21.41% for ketones (VOO3).

The interpretation of the sensory changes during storage by means of volatile compounds requires the study of the individual compounds, in particular of those that are odor-active. In a first stage, the content of all the individual compounds (Table 2) was studied during the storage experiment in order to identify which compounds underwent significant changes (*p*-value < 0.05) during the storage experiment and an ANOVA analysis was performed comparing concentration values from the months 0–3 vs. 24–27 (fresh vs. aged oils). Most of the volatile compounds (at least 75%) showed statistically significant changes in all the oils (Table 2). Overall, the storage time showed a great effect on the volatile compound’s concentration in all cases except for some alcohols, such as 2-methylbutan-1-ol. The relative standard deviation (RSD%) of the concentration values was calculated for those compounds presenting significant changes (*p*-value < 0.05) comparing the initial and the last sample stored in the experiment (0 and 27 months). The objective was to identify the compounds whose concentration varied in a greater extent. Table 3 shows the compounds whose concentration changed with a RSD% higher than 50% in the four VOOs. These compounds were different in each oil and the total number of compounds with RSD% > 50% per oil points out the oxidation state of them at the end of the experiment. These compounds show that VOO1 and VOO3 underwent a decrease in the concentration of the majority of the selected compounds, which have pleasant attributes, such as (*E*)-3-hexen-1-ol, which explains the loss of their initial positive attributes (median of the fruity attribute) during the storage (Figure 2). Sample VOO3 showed a large increase of the concentration of heptanal and (*E*)-2-heptenal, which are related to oily and oxidized aroma descriptors [1,31,35]. Finally, VOO2 and VOO4 were in an advanced oxidation state at the end of the storage, as it is pointed out by the increase of the content of the volatiles related to the rancid defect [36], such as nonanal, heptanal and hexanoic acid.

In a next step—and in order to gain a better understanding about which compounds have more influence on the virgin olive oil aroma—the odor activity value (OAV) of each volatile compound was determined at every month during the entire storage time for the different oils. This value results from the ratio of the concentration of the compound to its odor threshold [37]. Many of the compounds derived from the lipoxygenase (LOX) pathway, which contribute with a green and fruit aroma sensory descriptors [1], showed an OAV > 1 in all samples during the entire storage time, such as hexanal, (*E*)-2-hexenal, hexyl acetate, hexanol and (*Z*)-3-hexenyl acetate. The OAV of hexanal and (*E*)-2-hexenal were particularly high: they were in the ranges of 51.10–27.69 and 13.69–2.84, respectively.

Other compounds with unpleasant sensory descriptors showed a high OAV from the beginning of the storage, such as 1-octen-3-ol in VOO1 (135.94), VOO2 (47.57) and VOO4 (47.99), heptanoic acid in VOO3 (49.49) and (*E*)-2-decenal in VOO4 (255.44). 1-octen-3-ol provides moldy odor to the oil, whereas heptanoic acid and (*E*)-2-decenal are volatile markers of the rancid defect [31,38]. Other compounds related to the rancid defect also showed OAV > 1 but at lower extent, such as nonanal and (*E*)-2-heptenal. They were found at low concentrations although their odor thresholds were low enough (0.005 mg/kg and 0.15 mg/kg for (*E*)-2-heptenal and nonanal respectively) to have some impact on the sensory characteristics of the oil. Thus, their OAV in the fresh samples were 2.96–3.49 and 1.26–1.63 for (*E*)-2-heptenal and nonanal, respectively.

**Figure 3:**
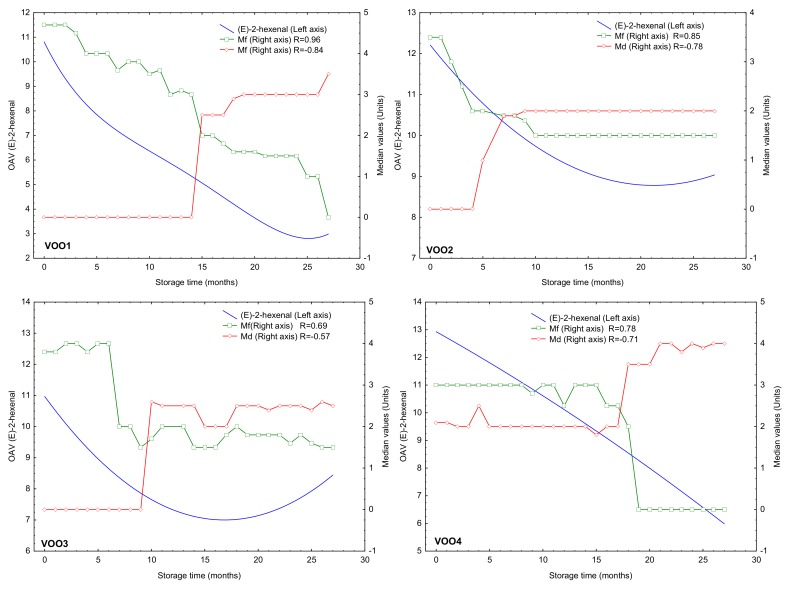
Median of the fruity attribute (Mf), median of defect (Md) and (*E*)-2-hexenal OAV (odor activity value) of the four virgin olive oils (VOO) during the storage experiment. The regression coefficients (R) between Mf and Md in relation to the OAV of (*E*)-2-hexenal are shown.

Once the compounds that underwent the most significant changes were identified (Table 3), all volatile compounds from Table 2 were studied to identify those whose concentration changes were better correlate to sensory changes. For this purpose, a correlation matrix was performed between the OAV of the volatile compounds (Table 2) and the results from the sensory assessment (medians of the fruity attribute and the defect) monthly obtained. Only (*E*)-2-hexanal, which exceeded its odor threshold in all samples during the storage experiment, showed a high correlation coefficient (0.70–0.96) with the median of the fruity attribute in the studied oils. Particularly, a strong correlation was found between these two variables in VOO1 (R = 0.96). This compound, which is considered as a freshness marker in vegetables oils [31,39], contributes with green and fruity attributes (Table 2). Figure 3 shows a double-y graph where the median of the fruity attribute and the OAV for (*E*)-2-hexenal are plotted per each VOO. Although (*E*)-2-hexenal concentration was reduced in a range of 1.16–3.33 mg/kg (Table 2), this compound with pleasant sensory descriptor showed an OAV higher than 1 during the entire storage time in all samples (OAV > 2.84 in all cases). Other compounds showing high correlation coefficients (>0.70) with median of the fruity attribute in some particular oils were (*Z*)-3-hexen-1-ol (R = 0.93 and 0.70 in VOO1 and VOO4, respectively) and (*E*)-2-hexen-1-ol (R = 0.96 in VOO1). The concentration of these two compounds is highly influenced by the stage of ripeness [1].

**Figure 4:**
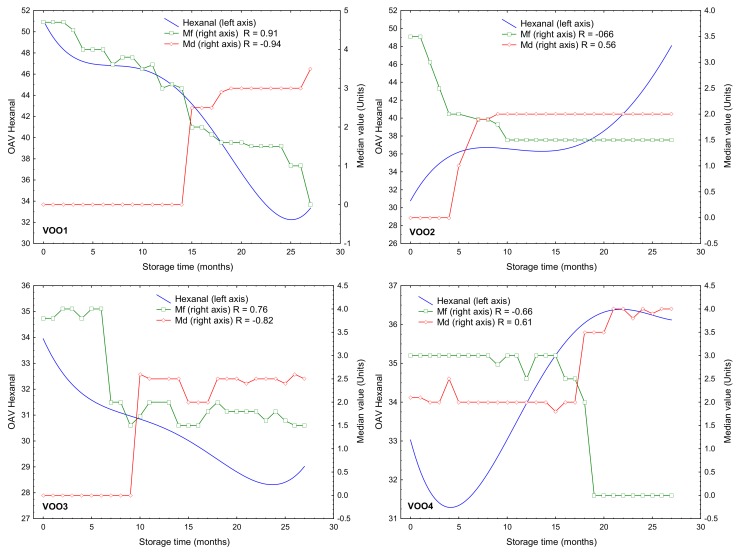
Double-y graph of the median of the fruity attribute (Mf), median of defect (Md) and the OAV (odor activity value) of hexanal for each virgin olive oil (VOO) during the storage experiment. The regression coefficients (R) between Mf and Md in relation to the OAV of hexanal are shown.

The OAV values of hexanal was also highly correlated (>0.70) with medians of the fruity attribute, although only in two oils. Thus, the correlation coefficients were 0.91 and 0.76 for VOO1 and VOO3, respectively, while a negative value (−0.66) was obtained for VOO2 and VOO4. Hexanal was not selected by ANOVA when comparing its concentration in the initial and last months of storage (Table 2) and it did not show a change in its concentration with a RSD% > 50% (Table 3). However, the study of its concentration and OAV during the storage under moderate conditions may provide useful information about the oxidation state of the samples since this compound is also produced during oxidation and it has an evident implication in virgin olive oil rancidity [3,40]. Figure 4 shows the OAV of hexanal and the medians of the fruity attribute and the defect represented on a double-y graph with respect to the storage time. Two kinds of trends of its OAV during the storage was observed. Thus, the hexanal OAV decreased in VOO1 and VOO3 during the storage; it shows the opposite trend in both Arbequina samples, VOO2 and VOO4, in which its concentration increased from the beginning of the storage. The decrease of OAV in VOO1 and VOO3 can be explained by a loss of hexanal present in the fresh oil originated from the LOX pathway while hexanal was later produced during oxidation at lower extent [1]. Figure 4 shows a similar trend in the decrease of median of the fruity attribute for VOO1—and in lesser degree—for VOO3, which explains the high correlation coefficients in these two cases. On the contrary, the opposite time-trends of OAV and median of the fruity attribute in VOO2 and VOO4 explain the negative value of the correlation coefficient (−0.66). However, the correlation coefficients when comparing OAV and median of defect in these two oils were positive although always below 0.8 (0.56 and 0.61 for VOO2 and VOO4, respectively) (Figure 4).

The increase of hexanal OAV and concentrations in VOO2 and VOO4 is explained by the decomposition reactions of hydroperoxides formed from the fatty acids [3,41], contributing to the off flavor of the sample with an intense greasy odor [42]. In fact, these off-flavors associated with rancidity is detected by the assessors at month 5 in VOO2 (sample VOO2-5m) and at month 19 in VOO4 (sample VOO4-19m) (Figure 2). These results indicate that VOO2 and VOO4 were in a more advanced oxidation state than the rest of the studied oils in the course of the storage experiment. This finding was not in agreement with the oxidative stability index from Rancimat method in the particular case of VOO4. Thus, VOO4 showed a high stability for Rancimat (53.60 h, the second most stable VOO) (Table 1). However, in the storage experiment, this VOO was undergraded to lampante category in the 19th month, while the rest of oils never were classified as lampante (VOO2, VOO3) or were classified as lampante later (VOO1 in 25th month). The evolution of PV values in VOO4 also shows a faster oxidation compared with the others, except for VOO1 (Figure 1). These results agree with the already reported relationship of hexanal with rancidity in aged oils [31,34,42].

**Figure 5:**
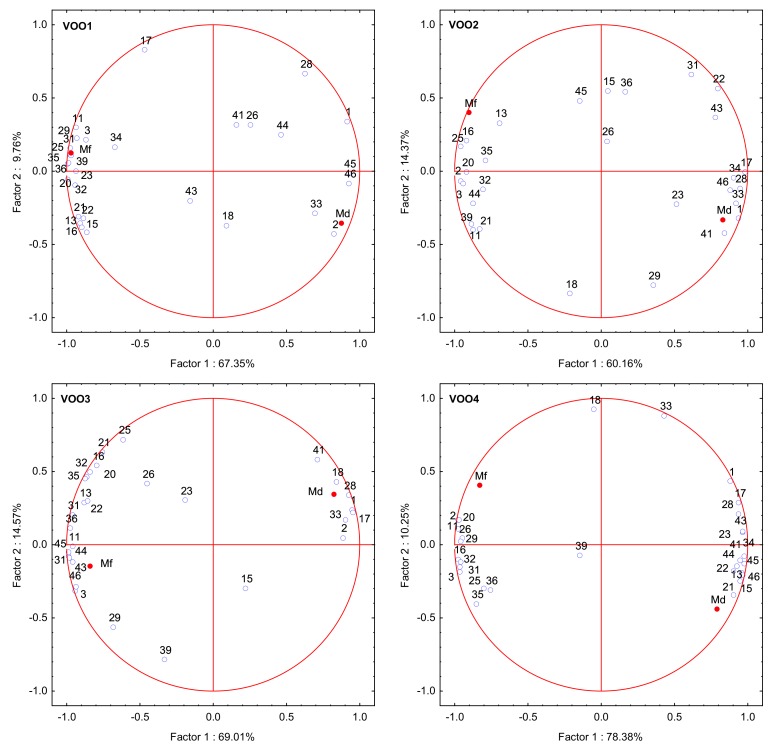
Principal component analysis plots (PCA) of volatile compounds that showed significant changes (*p* < 0.05) and RSD% > 50% in the storage experiment, the median of the fruity attribute (Mf) and the median of defect (Md) for the storage time. An individual plot was performed for each virgin olive oil (VOO). Note. Codes are indicated in Table 2.

In order to study the changes in the sensory characteristics and the volatile composition of the oils from a multivariate perspective, a principal component analysis (PCA) was carried out with the concentration values of the selected volatile compounds that showed a RSD% > 50% in the storage experiment (Table 3) and the medians of the fruity attribute and the defect. Figure 5 shows the resulting PCA plots for the 4 VOOs. The PCA plots show that the median of the fruity attribute and the median of defect were well separated by factor 1 in all VOOs, which were located in opposite quadrants. On the other hand, the plotted compounds were clustered by factor 1 according to their link to the median of the fruity attribute and the median of defect during the storage period. The median of the fruity attribute and the compounds associated to it were found in the left quadrant while the median of defect and the compounds related to it were located in the right quadrant. These results revealed than some compounds show a higher correlation with median of defect; this relationship was different depending on the oxidative state of the oil in the storage experiment. The PCA results show that the selected compounds that originated from the lipoxygenase pathway (Table 2), such as (*E*)-2-hexenal and (*Z*)-2-hexen-1-ol and the median of fruity attribute were plotted in the same quadrant in VOO1, VOO3 and VOO4. In the case of VOO2, however, the median of the fruity attribute appears to be only associated to (*Z*)-2-hexen-1-ol and (*Z*)-3-hexen-1-ol, but not with the rest of these compounds, which can be explained by the slight change in their concentrations in this oil during the storage time instead of a reduction in their concentration as in the other oils (Table 2). Furthermore, other compounds, such as butanal and butan-1-ol, were associated to the median of the fruity attribute in all VOOs, due to their concentration decrease during the storage, except for butan-1-ol in VOO2. This compound contributes with an aroma that is closer to the negative attributes (fatty and medicine for butan-1-ol) [43]. Other compounds contributing with negative attributes were plotted near the median of the fruity attribute, which is explained by the fact that their concentrations also decreased over time: methyl acetate in both VOO2 and VOO4, octan-2-one and 1-octen-3-one in VOO1 and nonanoic acid in VOO3.

Regarding the median of defect value, the PCA plots show an association of this median with the concentrations of octane, (*E*)-2-heptenal and (*E*)-2-decenal in all cases, these compounds being related with sensory defects and the two latter contributing with oxidized and fatty notes [1]. Moreover, nonanal and the median of defect were plotted in the same quadrant in VOO1, VOO2 and VOO3, which points out that the autooxidation process takes place during the storage experiment. However, this association was not observed in the PCA for VOO4, in which nonanal and median of defect were plotted in different quadrants despite this oil was the first one that downgraded to lampante at the end of the storage. These results may point out that the relationship of nonanal concentration and median of defect is more evident at earlier stages of oxidation as it is the case in VOO1, VOO2 and VOO3. On the other hand, a strong association was found for heptanal and the median of defect in VOO2, VOO3 and VOO4. In VOO1, this association was not found in the PCA plot, probably due to the fact that it was the oil that underwent the oxidation at lower extent in the first half of the storage experiment and the downgrading of category (from “extra virgin olive oil “to “virgin olive oil”) took place the latest (month 15) (Figure 2). Finally, the PCA plots showed that the hexanoic acid is related to the median of defect in the cases of VOO2 and VOO4.

In a second PCA, only the concentrations of the same volatile compounds (Table 3) were studied without including the medians of the fruity attribute and the defect in the data set. The objective was to check the score plot (samples) to study the changes of the volatile profile of the oils during the storage experiment with a multivariate perspective. Thus, Figure S1 shows a score plot per each VOO stored, in which the samples collected every month are represented against the factors. The score plot of VOO1 shows a change in the distribution of the samples from month 15 (VOO1-15m), which is the moment when this sample downgraded to “virgin olive oil” category (Figure 2). The score plot for VOO2 pointed out a change in the distribution of the monthly collected samples in the month 8 (VOO2-8m) when the assessors identified an incipient rancidity in this oil for the first time (Figure 2). Furthermore, in VOO3, the change of the trend of the samples was identified at month 10 (VOO3-10m) when assessors detected a winey-vinegary defect in this samples that caused an increase of 2.6 in its median of defect and, consequently, its downgrading to “virgin olive oil” category (Figure 2). Finally, in sample VOO4 two changes in the trend of the samples were observed in the score plot. Thus, a change in the distribution of the samples was identified at month 8 (VOO4-8m) and another change was detected at month 19 (VOO4-19m). In this last month, the oil downgraded to “lampante olive oil” according to the panel test (Figure 2). These results evidence that the selected compounds were able to explain the sensory changes identified by the assessor during the storage period.

The results obtained by Rancimat method (Table 1) before the storage experiment and the sensory assessment carried out during the storage (Figure 2) showed a different order in the stability of VOO. Thus, the order of oils from more to less stable according to Rancimat was VOO3, VOO4, VOO1 and VOO2, while this order was different according to the month in which a change of category takes place (from later to sooner): VOO1, VOO3, VOO2 and VOO4. That means that the results from Rancimat tests do not necessarily correlate with the actual stability in sensory terms. The evolution of PV values also pointed out VOO4 as one of the samples that faster was oxidized (Figure 1).

Finally, the results from the mesh cell-FTIR experiment, an innovative method to evaluate stability of the oils under moderate conditions, was examined with the aim of comparing these results with the actual stability of the oils according to the sensory changes. Mesh cell-FTIR allowed monitoring the chemical changes of the oils during an incubation time of 576 h under conditions of light and temperature (400 lx and 35 °C) that were closer to the real storage conditions compared with other accelerated tests that use high temperatures (>100 °C) [41]. The mesh cell-FTIR experiment was carried out with the fresh oils before starting the storage experiment. The spectral band assigned to the C=O stretching of unsaturated aldehydes (1685 cm^−1^) was monitored during the incubation time since it is related with secondary oxidation products and it rises during oxidation [6,20]. The maximum intensity for the aldehydes band was found in sample VOO2 and VOO4 with a value of 0.35 and 0.33 respectively. The other two oils showed lower values, these values being 0.28 for VOO3 and 0.25 for VOO1. These results revealed that both Arbequina VOOs were more susceptible to oxidation at moderate condition than the other two oils (VOO1 and VOO3). Considering this measurement, the order of stability (from high to low) was VOO1, VOO3, VOO4 and VOO2, which is close to the stability order according to the sensory testing (VOO1, VOO3, VOO2 and VOO4). Thus, mesh cell-FTIR pointed out that VOO1 was the most stable sample and, in fact, this sample changed of quality category the latest. On the contrary, Rancimat test pointed out that this sample was the second most unstable. These differences in results can be explained by the different mechanisms of oxidation that are involved depending on the conditions [44]. It illustrates the necessity of studying the VOO oxidative stability at moderate conditions and including light as a relevant variable.

**Figure 6:**
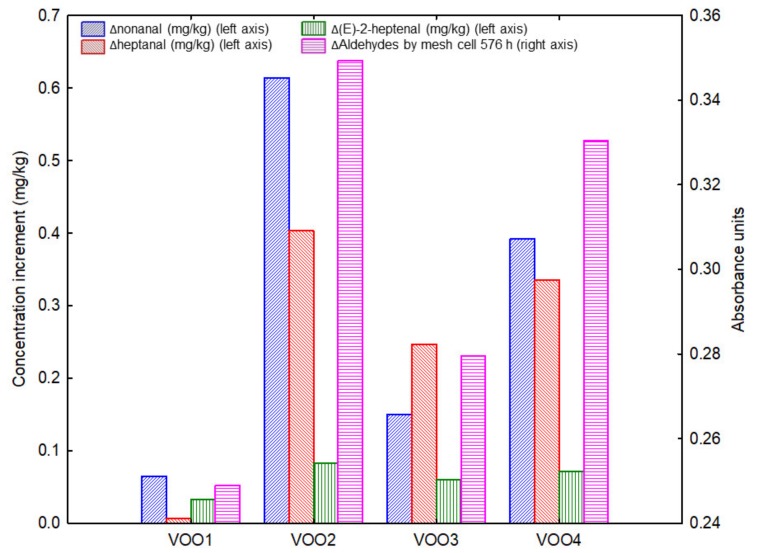
Double-y column graph in which the absorbance of the band assigned to aldehydes and the concentration increment (Δ) of nonanal, heptanal and (*E*)-2-heptenal are represented for the all studied virgin olive oils (VOOs) during the storage period.

In a farther study, with the aim of studying the ability of the mesh cell-FTIR band assigned to aldehydes to represent the changes of volatile composition of VOO during the storage, the results obtained by mesh cell-FTIR were compared with the concentration increment of the compounds associated to the rancid defect and that showed significant changes during the storage. The concentration increment was calculated using the final and initial concentrations of these compounds (showed in Table 2). Figure 6 shows a double-y column graph in which the mesh cell-FTIR absorbance of the band assigned to aldehydes and the concentration increment (mg/kg) of nonanal, heptanal and (*E*)-2-heptenal of each VOO during the storage period are shown. Although the concentration increment of the selected volatiles revealed more differences between the samples than the results obtained by mesh cell, they revealed the same order of oxidative stability of the samples.

## 3. Materials and Methods

### 3.1. Samples

Four monovarietal VOOs from the cultivars Picual, Hojiblanca and Arbequina (2 different samples of the last cultivar) provided by different producers were selected. These cultivars were chosen to cover different chemical compositions. The codes used to identify the VOOs and their respective cultivars were: VOO1, Hojiblanca; VOO2, Arbequina-1; VOO3, Picual; and VOO4, Arbequina-2. In order to guarantee that the samples VOO1–VOO4 were fresh at the beginning of the experiment, they were directly collected from the vertical centrifuge at the oil mills and then filtered to remove water. This time was considered as “time zero” and the storage experiment started just after collecting the samples.

VOOs was packaged in 27 transparent PET bottles of 500 mL—which are commonly used to bottle VOO—and they were hermetically sealed. The oil bottles were stored during 27 months in a compartment specially designed for it, where samples were exposed to light intensity of 1000 lx in 12 h light/dark cycles, simulating the conditions of a supermarket shelf under controlled conditions of temperature and humidity. The maximum and minimum of temperature and humidity were measured daily, being 29.7 °C–16.3 °C and 70%–21%, respectively. A bottle per oil was taken from the compartment monthly, analyzed and discarded afterwards. Thus, the analyses were carried out on VOOs from bottles newly opened. In order to identify the samples corresponding to each month, the number of each month and the letter “m” was added to the initial code (e.g., VOO1-5m means VOO of Hojiblanca cultivar after 5 months of storage).

### 3.2. Quality Parameters

The fatty acid composition and the trans fatty acid content were determined in the fresh samples (month 0) following the standard method (COI/T.20/Doc. No 33) [45].

Quality parameters were determined to confirm the quality category before starting the storage and to track their change during the experiment. These parameters were peroxide value (PV), free acidity (free fatty acids or FFA), which were determined by titration according to their respective standard methods (COI/T.20/Doc. No 35 and COI/T.20/Doc. No 34) [46,47]; and ultra-violet absorbance measured through the extinction coefficients (K_270_ and K_232_), which were determined according to the standard method (COI/T.20/Doc. No 19) [48].

### 3.3. Oil Stability Index (OSI)

The VOOs were analyzed to determine their oxidative stability by Rancimat method, also called oil stability index (OSI), before starting the storage experiment. OSI was determined according to the standard method AOCS Cd 12b-9 [49]. This method consists in heating samples at 100 °C while a continuous stream of air (20 L/h) is passing through the samples. The air is bubbled through a vessel with 60 mL of deionized water. The reported result is increase of the conductivity of the water due to the formation of volatile organic acids.

### 3.4. Mesh Cell-FTIR Analysis

Mesh cell-FTIR analyses were carried out with the fresh VOOs, following the method proposed by Tena et al. [6]. Aliquots of the fresh oils (20 μL) were deposited onto the mesh and they were stored at 35 °C and 400 lx. The spectral changes during the experiment were monitored daily during an incubation time of 576 h using a Bruker vertex 70 FTIR spectrometer (Bruker, Optics, Germany) equipped with a deuterated triglycine sulfate (DTGS) detector. The spectra were collected and manipulated with OPUS software version 7.2 (Bruker Optics, Ettlingen, Germany). The band assigned to the C=O stretching of unsaturated aldehydes (1685 cm^−1^) was selected to track the changes during incubation. The peak heights of this band were measured relative to a selected single-point baseline at 1576 cm^−1^ by implementing a macro programed on Omnic 7.3 (Thermo Electron Inc., Madison, WI, USA).

### 3.5. Determination of Volatile Compounds

The volatile compounds were determined by solid phase microextraction-gas chromatography (SPME-GC) including a preconcentration step carried out on a multipurpose sample autosampler (Gerstel, Mülheim an der Ruhr, Germany) in which temperature and time of the process were automatically controlled by Gerstel Maestro (v1.4) software (Gerstel GmbH et Co.KG, Mülheim an der Ruhr, Germany).

The oil sample (2 g) was placed in a 20 mL glass vial, tightly capped with polytetrafluoroethylene (PTFE) septum and left for 10 min at 40 °C to allow for the equilibration of the volatiles in the headspace. After the equilibration time, the septum covering each vial was pierced with a SPME needle and the fiber was exposed to the headspace for 40 min. A 1-cm StableFlex divinylbenzene/carboxen/polydimethylsiloxane (DVB/CAR/PDMS) composite SPME fiber (50/30 µm film thickness) was used (Supelco, Bellefonte, PA, USA). The fiber was previously conditioned following the instructions of the supplier.

The volatiles adsorbed by the fiber were thermally desorbed in the hot injection port of a 7820A gas chromatograph (Agilent Technologies, Madrid, Spain) with a flame ionization detector for 5 min at 260 °C, with the purge valve off (splitless mode). An Agilent J&W GC DB-WAX capillary column (60 m × 0.25 mm internal diameter, 0.25 µm coating) (Agilent Technologies, Santa Clara, CA, USA) was used. The carrier gas was hydrogen, at a flow rate of 1.5 mL/min. The oven temperature was held at 40 °C for 10 min and then programmed to rise 3 °C/min to a final temperature of 200 °C.

The identification of the volatiles was carried out with standards [29]. Additionally, the identification was checked by analyzing the samples with mass spectrometry (GC7820-MSD5975, Agilent Technology, Santa Clara, CA, USA) following the strategy described in previous works [38,50]. Thus, the information from mass spectra, and their comparison with standards in the FID chromatograms, and linear retention indexes (LRIs) were considered for a full identification. The quantification of volatiles was carried out by using 4-methyl-2-pentanol as internal standard and correcting the concentrations by the relative response factors. These factors were calculated following the procedure described by Oliver-Pozo et al. [51] using two ranges of concentration, 0.05–5.0 and 0.5–30 mg/kg.

The odor activity values (OAVs) [52,53] of the volatile compounds were calculated in all the monthly collected samples, in order to study the sensory influence of each compound on the total aroma of VOO and its evolution during the storage.

### 3.6. Sensory Assessment

The organoleptic assessment of the olive oil samples was carried out monthly by the accredited panel of Instituto de la Grasa (UNE-EN-ISO/IEC 17025) (Seville, Spain) [54] using the standard method COI/T.20/Doc. No 15/Rev.10 [55]. Eight-twelve trained assessors qualified the samples by odor descriptors and established if the samples had any defects, to determine the progression of off-flavors during the storage under moderate conditions.

The results monthly generated from the sensory assessment were based on the calculation of the medians of the fruity attribute and the defect for the four stored VOOs. This test provided a sequential information about the sensory characteristics of the samples and allowed identifying changes in the category of the oils and in their sensory profile.

### 3.7. Determination of Phenol Content

The method for the determination of phenol composition was based on the method described by Aparicio-Ruiz et al. [56]. The sample (2.5 g) was solved in 6 mL of hexane together with *p*-hydroxyphenylacetic (0.12 mg/mL) and *o*-coumaric (0.01 mg/mL) as internal standards. The phenolic fraction was extracted with methanol by solid phase extraction using diol-bonded phase cartridges. The extracted phenolic fraction was concentrated and injected in the HPLC system (Agilent Technologies 1200, Waghaeusel–Wiesental, Germany), equipped with a diode array detector. The column was a LiChrospher 100RP-18 column (4.0 mm i.d. × 250 mm; 5 µm, particle size) (Merck KGaA, Darmstadt, Germany) maintained at 30 °C. The gradient elution, at a flow rate of 1.0 mL/min, was achieved by using a mixture of water/ortho-phosphoric acid (99.5:0.5 *v*/*v*) (solvent A) and methanol/acetonitrile (50:50 *v*/*v*) (solvent B). The change in solvent gradient was programed as follows: from 95% (A)-5% (B) to 70% (A)-30% (B) in 25 min; 65% (A)-35% (B) in 10 min; 60% (A)-40% (B) in 5 min; 30% (A)-70%(B) in 10 min and 100% (B) in 5 min, followed by 5 min of maintenance. The chromatographic signals were obtained at 235, 280 and 335 nm. The quantification of the phenols, cinnamic acid and lignans was carried out at 280 nm using p-hydroxyphenylacetic acid as internal standard. The quantification of flavones was done at 335 nm by using *o*-coumaric acid as internal standard. The response factors and recoveries were based on the procedure developed by Mateo et al. (2001) [57].

### 3.8. Statistical Analysis

The STATISTICA 8 package (Statsoft, Tulsa, OK, USA) was used to carry out the statistical analysis. A one-way ANOVA analysis was performed comparing concentration values from the months 0–3 vs. 24–27 (fresh vs. aged oils). Significance was accepted when *p* < 0.05.

Principal component analysis (PCA) was carried out on the concentration values of volatile compounds and the medians of defect and fruity attribute to explore the data from a multivariate perspective and to support the relationship between compounds and the observed changes in the sensory assessment.

## 4. Conclusions

This study shows the complexity of predicting the shelf-life of VOO overall when the sensory quality is considered as a relevant criterion in addition to physico-chemical parameters. Thus, the sensory defects that can appear during storage can be detected by consumers with a resulting refusal of the product. On the other hand, this change of quality can be marked enough to result in a downgrading of category (from “extra virgin olive oil” to “virgin olive oil” and even to “lampante olive oil”), with the consequent non-conformity result when the oil category is checked by a panel test. This change of quality is explained by the changes in the concentrations of volatile compounds during storage. Thus, while the compounds contributing with positive sensory attributes (C6 and C5 compounds) reduce their concentration, those compounds coming from oxidation (mainly aldehydes and acids) increase their concentration. The panel tests revealed that the reduction of the compounds contributing to positive notes can result in the detection of fermentative sensory defects (e.g., winey-vinegary) that the oil already contained but they were masked by the intense green aroma. The oil stability studied by Rancimat at the beginning of the storage produced results that disagreed with the actual sensory changes detected in the storage experiment. The stability studied by mesh-cell FTIR revealed that the band assigned to aldehydes permitted to establish a stability order of the oils that was closer to the order of stability according to the sensory changes (determined by panel test). This result underscores the importance of considering light and moderate temperatures when studying the oil stability in order to avoid unexpected quality-category downgrading. The results also showed that the changes on sensory characteristics and volatile profile followed a different trend depending on the studied oil and there is not a uniform change rate that could be stablished for all the oils.

## Figures and Tables

**Table 1:** Quality parameters, together with the quality criteria of extra virgin olive oil (EVOO) according to EC regulation No 2568/91 and updates; stability index, fatty acid composition and total phenol content in the fresh virgin olive oils, before of the bottling. The virgin olive oil codes, dates of the extraction and the cultivars are shown.

Chemical Parameters		Virgin Olive Oil Codes				Quality Criteria (EVOO)
		VOO1	VOO2	VOO3	VOO4	
Cultivar Extraction Date (dd/mm/yy)		Hojiblanca	Arbequina	Picual	Arbequina	
		31/10/15	3/12/2015	12/11/2015	20/11/2015	
Organoleptic characteristics	Mf ^a^	4.7	3.5	3.8	3	>0
	Md ^b^	0	0	0	2.1	=0
Free fatty acid (% m/m of oleic acid)		0.15	0.21	0.20	0.22	≤0.8
Peroxide value (PV) (meq O_2_/kg)		4.30	5.13	3.63	4.80	≤20
K_232_ $(K_{1\mathrm{cm}}^{1\%}$)		1.52	1.87	1.72	1.84	≤2.50
K_270_ $(K_{1\mathrm{cm}}^{1\%}$)		0.06	0.19	0.04	0.18	≤0.22
Total phenols (mg/kg)		226.71	338.90	534.82	468.10	
Oil stability index (OSI) at 100 °C (h)		38.71	22.95	82.80	53.60	
C16:0 (%)		14.30	15.46	10.79	16.02	
C16:1 (%)		0.93	1.77	1.19	1.87	
C17:0 (%)		0.18	0.11	0.04	0.15	
C17:1 (%)		0.29	0.24	0.08	0.27	
C18:0 (%)		2.56	1.95	2.49	1.91	
C18:1 (%)		72.38	66.71	80.28	65.93	
C18:2 (%)		7.29	12.22	3.45	12.36	
C18:3 (%)		1.08	0.66	0.82	0.65	
C20:0 (%)		0.47	0.40	0.41	0.39	
C20:1 (%)		0.31	0.30	0.28	0.28	
C22:0 (%)		0.14	0.13	0.12	0.12	
C24:0 (%)		0.09	0.07	0.065	0.06	
C12:0, C14:0, tC18:1, tC18:2, tC18:3 (%)		<0.01	<0.01	<0.01	<0.01	
∑SFA (%)		17.73	18.11	13.91	18.64	
∑ MUFA (%)		73.90	69.01	81.82	68.35	
∑ PUFA (%)		8.37	12.88	4.27	13.01	
∑ UFA (%)		82.27	81.89	86.09	81.36	

^a^ Median of the fruity attribute. ^b^ Median of defect.

**Table 2:** Volatile compounds identified in the studied virgin olive oils, with their codes, their concentrations (mg/kg) at two different moments (before and after the storage experiment), the odor threshold and the sensory attributes of each volatile compound.

		VOO1		VOO2		VOO3		VOO4			
		Hojiblanca		Arbequina		Picual		Arbequina		Odor Threshold (mg/kg)	Aroma Sensory Descriptor
CODE	Months of Storage	0	27	0	27	0	27	0	27		
**1**	Octane	0.33 ^a^	4.24 ^a,b^	1.06 ^a,b^	12.87 ^a,b^	0.65 ^a^	13.95 ^a,b^	2.012 ^a,b^	14.13 ^a,b^	0.94	Sweet, alkane
**2**	Methyl acetate	0.51 ^a,b^	0.59 ^a,b^	1.28 ^a,b^	0.89 ^a,b^	0.13 ^a^	0.18 ^a^	1.03 ^a,b^	0.49 ^a,b^	0.20	Solvent, fruit
**3**	Butanal	0.05 ^a,b^	0.03 ^a,b^	0.05 ^a,b^	0.02 ^a,b^	0.07 ^a,b^	0.03 ^a,b^	0.04 ^a,b^	0.03 ^a,b^	0.08	Green, pungent
**4**	Ethyl acetate	0.70 ^a^	0.61 ^a^	1.11 ^a,b^	1.01 ^a,b^	0.16 ^a^	0.19 ^a^	1.59 ^a,b^	1.00 ^a,b^	0.94	Sticky, sweet
**5**	Butan-2-one	0.48 ^a^	0.37 ^a^	0.29 ^a^	0.28 ^a^	0.55 ^a^	0.41 ^a^	0.12 ^a^	0.10 ^a^	40.00	Ethereal, fruit
**6**	2-methylbutanal	0.04 ^a,b^	0.03 ^a,b^	0.15 ^a,b^	0.11 ^a,b^	0.05 ^a,b^	0.08 ^a,b^	0.16 ^a,b^	0.12 ^a,b^	0.005	Malty
**7**	3-methylbutanal	0.02 ^a,b^	0.03 ^a,b^	0.06 ^a,b^	0.11 ^a,b^	0.04 ^a,b^	0.08 ^a,b^	0.09 ^a,b^	0.05 ^a,b^	0.005	Malty
**8**	Ethanol	19.63 ^a^	15.59 ^a^	12.17 ^a^	5.96 ^a^	21.09 ^a^	15.67 ^a^	15.88 ^a^	9.45 ^a^	30.00	Alcohol
**9**	Ethyl propanoate	0.76 ^a,b^	0.64 ^a,b^	0.32 ^a,b^	0.26 ^a,b^	0.24 ^a,b^	0.29 ^a,b^	0.10 ^a^	0.05 ^a^	0.10	Fruit, strong
**10**	3-pentanone	4.83 ^a,b^	4.04 ^a,b^	2.41 ^b^	2.20 ^b^	3.46 ^a,b^	2.82 ^a,b^	2.23 ^a,b^	1.60 ^a,b^	70.00	Sweet, fruit
**11**	Butan-2-ol	0.07 ^a^	0.04 ^a^	0.09 ^a^	0.05 ^a^	0.09 ^a^	0.04 ^a^	0.06 ^a^	0.02 ^a^	0.15	Winey
**12**	Hexanal ^c^	3.83 ^a,b^	2.33 ^a,b^	2.08 ^a,b^	3.75 ^a,b^	2.62 ^a,b^	2.19 ^a,b^	2.41 ^a,b^	2.72 ^a,b^	0.08	Green-sweet
**13**	2-methylpropan-1-ol	0.12 ^a^	0.02 ^a^	0.04	0.01	0.02 ^a^	0.01 ^a^	0.01 ^a^	0.02 ^a^	1.00	Wine, solvent
**14**	1-penten-3-ol	0.96 ^a,b^	0.65 ^a,b^	0.44 ^a,b^	0.31 ^a^	0.51 ^b^	0.52 ^b^	0.40 ^a^	0.31 ^a^	0.40	Pungent, butter
**15**	(*E*)-2-pentenal	0.46 ^a,b^	0.23 ^a^	0.16	0.16	0.26	0.30 ^b^	0.12 ^a^	0.31 ^a,b^	0.30	Green, apple
**16**	Butan-1-ol	0.30 ^a^	0.07 ^a^	0.01 ^a^	0.01 ^a^	0.10	0.01	0.04 ^a^	0.03 ^a^	0.40	Fatty–medicine
**17**	Heptanal	0.10	0.09	0.06 ^a^	0.47 ^a^	0.05 ^a^	0.30 ^a^	0.08 ^a^	0.42 ^a^	0.50	Oily, fatty, Woody
**18**	2-methylbutan-1-ol	0.01	0.01	Nd ^d^	Nd ^d^	0.01 ^a^	0.05 ^a^	0.02	0.03	0.30	Winey, spicy
**19**	3-methylbutan-1-ol	0.03	0.03	0.04 ^a^	0.04 ^a^	0.02 ^a^	0.01 ^a^	0.08	0.06	0.10	Woody, whiskey
**20**	(*E*)-2-hexenal ^c^	4.53 ^a,b^	1.21 ^a,b^	5.03 ^a,b^	3.87 ^a,b^	5.81 ^a,b^	3.31 ^a,b^	5.71 ^a,b^	2.57 ^a,b^	0.42	Green, apple-like
**21**	Octan-3-one	0.08 ^a^	0.03 ^a^	0.04 ^a^	0.01 ^a^	0.15	0.09	0.02 ^a^	0.08 ^a^	0.75	Herb, butter
**22**	Pentanol	0.03 ^a^	0.01 ^a^	0.01 ^a^	0.06 ^a^	0.06	0.03	0.01 ^a^	0.01 ^a^	0.47	Fruity
**23**	1-octen-3-one	0.17 ^a^	0.07 ^a^	0.16	0.43	0.11	0.11	0.10 ^a^	0.20 ^a^	0.01	Mushroom, mold
**24**	Hexyl acetate ^c^	1.87 ^b^	1.96 ^b^	0.85	0.75	1.69 ^a,b^	1.52 ^a,b^	0.89 ^a^	0.70 ^a^	1.04	Green, fruity, sweet
**25**	Octan-2-one	0.08 ^a^	0.03 ^a^	0.02 ^a^	0.01 ^a^	0.12 ^b^	0.04	0.02 ^a^	0.01 ^a^	0.51	Mold, green
**26**	Octanal	0.07	1.38 ^b^	0.42 ^b^	0.98 ^b^	1.01 ^b^	1.00 ^b^	0.55 ^a,b^	0.31 ^a,b^	0.32	Fatty, sharp, citrus-like
**27**	(Z)-3-hexenyl acetate ^c^	0.63 ^a^	0.31 ^a^	0.56 ^a^	0.50 ^a^	1.73 ^b^	0.98	0.48 ^a^	0.31 ^a^	0.20	Green
**28**	(*E*)-2-heptenal	0.08 ^a,b^	0.05 ^a,b^	0.02 ^a,b^	0.10 ^a,b^	0.02 ^a,b^	0.08 ^a,b^	0.02 ^a,b^	0.09 ^a,b^	0.005	Oxidized, tallow
**29**	6-methyl-5-hepten-2-one	0.01 ^a^	0.01 ^a^	0.01	0.02	0.02 ^a^	0.02 ^a^	0.01 ^a^	0.01 ^a^	1.00	Pungent, green
**30**	Hexanol ^c^	3.56 ^a,b^	3.54 ^a,b^	1.57 ^a,b^	1.41 ^a,b^	1.65 ^a,b^	1.43 ^a,b^	1.08 ^a,b^	0.88 ^a,b^	0.40	Fruit, banana, soft
**31**	(*E*)-3-hexen-1-ol ^c^	0.46 ^a^	0.06 ^a^	0.10 ^a^	0.11 ^a^	0.21 ^a^	0.09 ^a^	0.05 ^a^	0.03 ^a^	1.00	Green
**32**	(Z)-3-hexen-1-ol ^c^	1.10 ^a^	0.45 ^a^	0.16 ^a^	0.12 ^a^	0.29	0.16	0.05 ^a^	0.04 ^a^	1.10	Green
**33**	Nonanal	0.22 ^a,b^	0.29 ^a,b^	0.19 ^a,b^	0.80 ^a,b^	0.24 ^a,b^	0.39 ^a,b^	0.22 ^a,b^	0.61 ^a,b^	0.15	Fatty, waxy, pungent
**34**	1-octen-3-ol	0.14 ^a,b^	0.04 ^a,b^	0.05 ^a,b^	0.08 ^a,b^	0.14 ^a,b^	0.04 ^a,b^	0.05 ^a,b^	0.09 ^a,b^	0.001	Moldy, earthy
**35**	(*E*)-2-hexen-1-ol ^c^	0.83 ^a^	0.23 ^a^	0.50 ^a^	0.37 ^a^	0.85 ^a^	0.44 ^a^	0.25 ^a^	0.19 ^a^	5.00	Green grass, leaves
**36**	(Z)-2-hexen-1-ol ^c^	1.43 ^a^	0.43 ^a^	0.29 ^a^	0.30 ^a^	0.59 ^a^	0.23 ^a^	0.16 ^a^	0.13 ^a^	1.00	Green
**37**	Acetic acid	1.63 ^a,b^	1.56 ^a,b^	5.16 ^b^	6.36 ^b^	2.94 ^a,b^	3.78 ^a,b^	0.63 ^b^	0.79 ^b^	0.50	Sour, vinegary
**38**	Propanoic acid	0.14 ^a^	0.24 ^a^	0.18 ^a^	0.19 ^a^	0.13 ^a^	0.20 ^a^	0.10	0.13	0.72	Pungent, sour
**39**	Butanoic acid	0.47 ^a^	0.23 ^a^	0.13 ^a^	0.06 ^a^	0.25	0.24	0.25	0.15	0.65	Rancid, cheese
**40**	2-methylpropanoic acid	0.08 ^a^	0.04 ^a^	0.07 ^a^	0.05 ^a^	0.10 ^a^	0.06 ^a^	0.06 ^a^	0.04 ^a^	-	Butter, cheese, rancid
**41**	(*E*)-2-decenal	0.13 ^b^	0.07 ^b^	0.12 ^a,b^	0.80 ^a,b^	2.75 ^a,b^	3.37 ^a,b^	2.55 ^a,b^	3.47 ^a,b^	0.01	Painty, fishy, fatty
**42**	Pentanoic acid	0.24	0.25	0.13 ^a^	0.24 ^a^	0.29	0.15	0.11 ^a^	0.20 ^a^	0.60	Unpleasant, pungent
**43**	Hexanoic acid	1.50 ^b^	1.78 ^b^	0.57 ^a^	1.22 ^a,b^	2.51 ^a,b^	0.57 ^a^	0.63 ^a^	1.23 ^a,b^	0.70	Pungent, rancid
**44**	Heptanoic acid	1.27 ^a,b^	2.97 ^a,b^	3.99 ^a,b^	1.52 ^a,b^	4.96 ^a,b^	1.16 ^a,b^	1.15 ^a,b^	4.00 ^a,b^	0.10	Rancid, fatty
**45**	Octanoic acid	1.07 ^a^	2.62 ^a^	1.77 ^a^	1.57 ^a^	4.47 ^a^	1.33 ^a^	1.03 ^a^	2.14 ^a^	3.00	Oily, fatty
**46**	Nonanoic acid	0.02 ^a^	0.08 ^a^	0.02 ^a^	0.05 ^a^	0.12 ^a^	0.02 ^a^	0.02 ^a^	0.05 ^a^	-	Fat, must, sweat, sour
	Total volatiles	55.00	49.54	43.94	50.47	63.29	57.91	42.65	49.36		

^a^ Significant difference (*p* > 0.05) in the concentration of the compound at the beginning and end of the storage; ^b^ the concentration of the compound exceeds its odor threshold (Odor Activity Value, OAV >1). ^c^ Compounds derived from the lipoxygenase (LOX) pathway. ^d^ Not detected.

**Table 3:** Selected volatile compounds that showed statistically significant changes (*p*-value < 0.05) and RSD% higher than 50% during the storage at moderate conditions.

VOO1		VOO2	
Compound	RSD%	Compound	RSD%
Octane ^a^	120.82	Octane	119.81
Butan-2-ol	50.00	Butanal	58.59
2-methylpropan-1-ol	93.90	2-methylpropan-1-ol	75.00
Butan-1-ol	90.08	Butan-1-ol	69.05
(*E*)-2-hexenal	82.03	Heptanal ^a^	107.53
Octan-3-one	70.06	Octan-3-one	92.07
Pentanol	78.22	Octan-2-one	68.64
Octan-2-one	74.39	Octanal ^a^	56.58
Octanal ^a^	128.77	1-octen-3-one ^a^	64.17
1-octen-3-one	59.92	(*E*)-2-heptenal ^a^	99.97
(*E*)-2-heptenal ^a^	71.08	6-methyl-5-hepten-2-one ^a^	64.13
6-methyl-5-hepten-2-one	61.66	Nonanal ^a^	87.46
(*E*)-3-hexen-1-ol	107.40	Butanoic acid	51.36
(*Z*)-3-hexen-1-ol	59.21	(*E*)-2-decenal ^a^	104.44
1-octen-3-ol	72.03	Hexanoic acid ^a^	50.98
(*E*)-2-hexen-1-ol	79.88	Heptanoic acid	63.61
(*Z*)-2-hexen-1-ol	76.50	Nonanoic acid ^a^	78.26
Heptanoic acid ^a^	56.85		
Octanoic acid ^a^	59.30		
Nonanoic acid ^a^	94.12		
**VOO3**		**VOO4**	
**Compound**	**RSD%**	**Compound**	**RSD%**
Octane ^a^	128.80	Octane ^a^	106.09
Butanal	56.41	Methyl acetate	50.03
Butan-2-ol	64.18	Butan-2-ol	80.69
2-methylpropan-1-ol	71.54	2-methylpropan-1-ol ^a^	64.37
Butan-1-ol	118.65	(*E*)-2-pentenal ^a^	62.07
Heptanal ^a^	100.26	Heptanal ^a^	94.48
2-methylbutan-1-ol ^a^	99.86	(*E*)-2-hexenal	53.76
Octan-2-one	77.44	Octan-3-one ^a^	79.59
(*E*)-2-heptenal ^a^	95.01	Pentanol ^a^	53.96
6-methyl-5-hepten-2-one	97.30	(*E*)-2-heptenal ^a^	95.28
(*E*)-3-hexen-1-ol	58.40	6-methyl-5-hepten-2-one	80.14
1-octen-3-ol	74.51	Nonanal ^a^	66.11
(*Z*)-2-hexen-1-ol	63.51	Heptanoic acid ^a^	78.27
Hexanoic acid	88.83	Octanoic acid ^a^	49.46
Heptanoic acid	87.90	Nonanoic acid ^a^	80.15
Octanoic acid	76.55		
Nonanoic acid	105.60		

^a^ Compounds that underwent an increase of their concentrations during the storage.

## Supplementary Materials

The following are available online: Figure S1. PCA score plots of cases (monthly collected samples) calculated using the volatile compounds which showed significant changes (p<0.05) and RSD%>50% during the storage time. The numbers indicate the month when the sample was collected and analyzed in the storage experiment. Table S1. Concentration of volatile compounds (mg/kg) in VOO1 during the storage experiment. Table S2. Concentration of volatile compounds (mg/kg) in VOO2 during the storage experiment. Table S3. Concentration of volatile compounds (mg/kg) in VOO3 during the storage experiment. Table S4. Concentration of volatile compounds (mg/kg) in VOO4 during the storage experiment. Table S5. Concentration of the chemical series of the volatile compounds identified in the oils (mg/kg) in two different moments (before and after the storage experiment).
