# Supplementary material for: Tracking Sensory Characteristics of Virgin Olive Oils During Storage: Interpretation of Their Changes from a Multiparametric Perspective
Source: Molecules. 2020 Apr 7;25(7):1686. doi: 10.3390/molecules25071686 (PMC7180626; doi:10.3390/molecules25071686)
Supplement: Supplementary file 1 [file molecules-25-01686-s001.pdf]

SUPPLEMENTARY MATERIAL

Tracking sensory characteristics of virgin olive oils during storage: interpretation of their changes from a multiparametric perspective.

Ana Lobo-Prieto[1], Noelia Tena[2], Ramón Aparicio-Ruiz[2], María T. Morales[2], Diego L. García-González[1,*]

[1] Instituto de la Grasa (CSIC), Ctra. de Utrera, km. 1, Campus Universitario Pablo de Olavide - building 46, 41013 - Sevilla, Spain; ana.lobo@ig.csic.es (A.L.-P.)

[2] Department of Analytical Chemistry, Faculty of Pharmacy, University of Seville, Prof. García González, 2, 41012, Seville, Spain; noelia.tena@ig.csic.es (N.T.); aparicioruiz@cica.es (R.A.-R.); tmorales@us.es (M.T.M.)

* Correspondence: dlgarcia@ig.csic.es; Tel.: +34-954-611-550 (D.L.G.-G.)

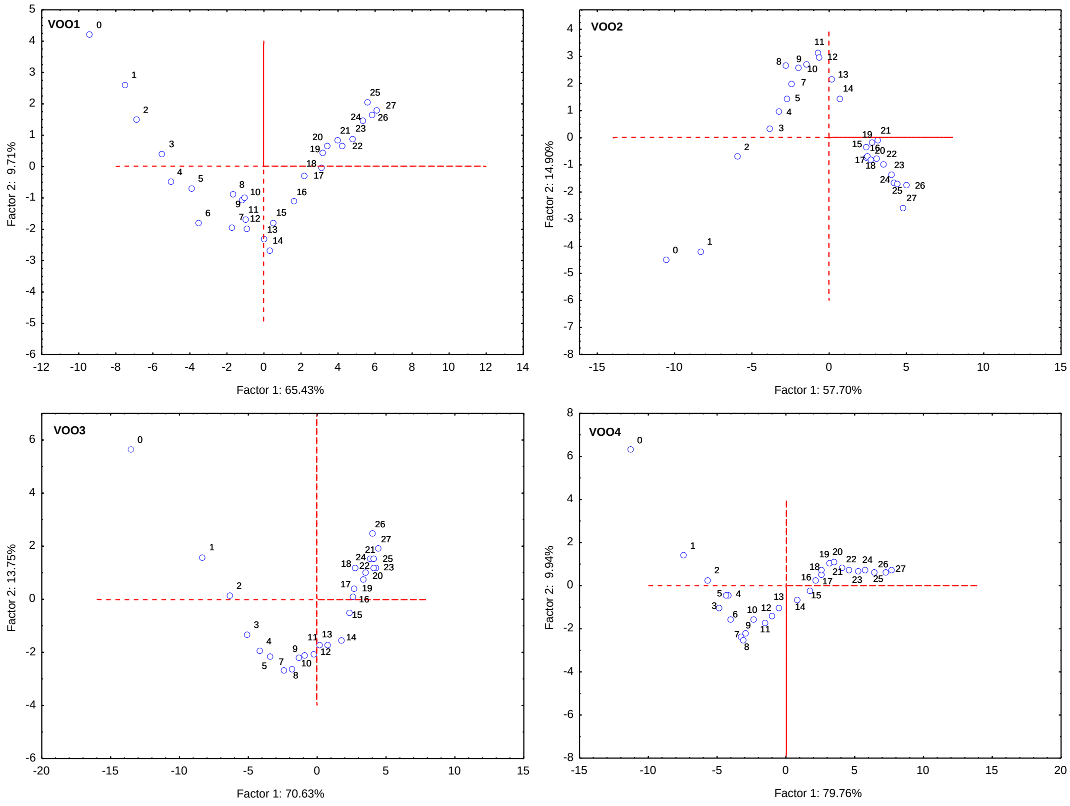

Figure S1. PCA score plots of cases (monthly collected samples) calculated using the volatile compounds which showed significant changes ($p<0.05$) and RSD%>50% during the storage time. The numbers indicate the month when the sample was collected and analyzed in the storage experiment.

Table S1. Concentration of volatile compounds (mg/kg) in VOO1 during the storage experiment.

**Months of storage**	**0**	**3**	**6**	**9**	**12**	**15**	**18**	**21**	**24**	**27**
Octane	0.33	1.68	2.63	3.05	3.55	3.55	3.63	4.06	4.12	4.24
Methyl acetate	0.51	0.49	0.45	0.51	0.50	0.51	0.56	0.56	0.57	0.59
Butanal	0.05	0.05	0.05	0.05	0.05	0.05	0.05	0.04	0.04	0.03
Ethyl acetate	0.70	0.68	0.63	0.64	0.62	0.61	0.61	0.63	0.61	0.61
Butan-2-one	0.48	0.43	0.41	0.42	0.41	0.41	0.39	0.41	0.38	0.37
2-methylbutanal	0.04	0.04	0.03	0.04	0.04	0.04	0.03	0.04	0.04	0.03
3-methylbutanal	0.02	0.02	0.01	0.02	0.01	0.01	0.02	0.02	0.02	0.03
Ethanol	19.63	18.20	17.84	18.05	16.96	17.17	15.93	16.52	15.53	15.59
Ethyl propanoate	0.76	0.70	0.65	0.62	0.65	0.64	0.70	0.66	0.65	0.64
3-pentanone	4.83	4.88	4.58	4.40	4.54	4.40	4.33	4.26	3.95	4.04
Butan-2-ol	0.07	0.08	0.08	0.06	0.06	0.06	0.04	0.04	0.03	0.04
Hexanal[c]	3.83	3.63	3.28	3.43	3.31	2.99	2.71	2.58	2.56	2.33
2-methylpropan-1-ol	0.12	0.08	0.05	0.04	0.05	0.05	0.04	0.03	0.02	0.02
1-penten-3-ol	0.96	0.94	0.83	0.82	0.81	0.76	0.75	0.72	0.65	0.65
(E)-2-pentenal	0.46	0.38	0.30	0.26	0.28	0.29	0.27	0.25	0.23	0.23
Butan-1-ol	0.30	0.23	0.14	0.11	0.10	0.11	0.08	0.07	0.07	0.07
Heptanal	0.10	0.14	0.15	0.14	0.16	0.14	0.11	0.10	0.09	0.09
2-methylbutan-1-ol	0.01	0.01	0.01	nd	nd	0.01	0.01	0.01	0.01	0.01
3-methylbutan-1-ol	0.03	0.03	0.03	0.03	0.03	0.03	0.03	0.03	0.03	0.03
(E)-2-hexenal	4.53	3.68	3.07	2.52	2.88	2.02	1.62	1.46	1.27	1.21
Octan-3-one	0.08	0.05	0.04	0.04	0.04	0.04	0.03	0.03	0.03	0.03
Pentanol	0.03	0.02	0.01	0.01	0.01	0.01	0.01	0.01	0.01	0.01
1-octen-3-one	0.17	0.14	0.10	0.11	0.13	0.11	0.08	0.07	0.07	0.07
Hexyl acetate	1.87	1.93	1.80	1.91	1.95	1.93	2.03	1.98	1.96	1.96
Octan-2-one	0.08	0.07	0.06	0.06	0.06	0.05	0.04	0.03	0.03	0.03
Octanal	0.07	1.76	1.26	1.42	1.44	1.32	1.40	1.63	1.63	1.38
(Z)-3-hexenyl acetate	0.63	0.54	0.54	0.42	0.50	0.55	0.37	0.36	0.31	0.31
(E)-2-heptenal	0.08	0.03	0.05	0.04	0.06	0.07	0.05	0.05	0.05	0.05
6-methyl-5-hepten-2-one	0.01	0.01	0.01	0.01	0.01	0.01	0.01	0.01	0.01	0.01
Hexanol[c]	3.56	3.64	3.60	3.40	3.46	3.42	3.69	3.57	3.56	3.54
(E)-3-hexen-1-ol	0.46	0.42	0.38	0.30	0.31	0.30	0.22	0.12	0.07	0.06
(Z)-3-hexen-1-ol	1.10	0.83	0.90	0.68	0.61	0.65	0.59	0.56	0.45	0.45
Nonanal	0.22	0.18	0.23	0.18	0.20	0.22	0.25	0.24	0.26	0.29
1-octen-3-ol	0.14	0.11	0.12	0.09	0.10	0.10	0.08	0.07	0.06	0.04
(E)-2-hexen-1-ol	0.83	0.73	0.67	0.53	0.54	0.48	0.39	0.30	0.24	0.23
(Z)-2-hexen-1-ol	1.43	1.37	1.17	0.95	0.93	0.92	0.63	0.56	0.44	0.43
Acetic acid	1.63	1.74	1.96	2.18	2.19	1.97	1.81	1.52	1.54	1.56
Propanoic acid	0.14	0.15	0.15	0.15	0.17	0.21	0.24	0.22	0.22	0.24
Butanoic acid	0.47	0.45	0.37	0.37	0.37	0.37	0.32	0.26	0.24	0.23
2-methylpropanoic acid	0.08	0.07	0.06	0.07	0.07	0.06	0.05	0.05	0.04	0.04
(E)-2-decenal	0.13	0.12	0.08	0.12	0.17	0.23	0.19	0.16	0.12	0.07
Pentanoic acid	0.24	0.25	0.24	0.21	0.21	0.23	0.29	0.28	0.26	0.25
Hexanoic acid	1.50	2.06	1.70	1.36	1.77	1.71	1.68	1.71	1.77	1.78
Heptanoic acid	1.27	1.96	1.86	1.69	2.00	3.20	3.18	2.04	2.15	2.97
Octanoic acid	1.07	1.15	1.70	1.62	1.79	2.10	2.38	2.12	2.35	2.62
Nonanoic acid	0.02	0.02	0.01	0.04	0.05	0.07	0.07	0.07	0.08	0.08

Note: nd, not detected.

Table S2. Concentration of volatile compounds (mg/kg) in VOO2 during the storage experiment.

**Months of storage**	**0**	**3**	**6**	**9**	**12**	**15**	**18**	**21**	**24**	**27**
Octane	1.06	8.08	10.43	10.44	11.19	11.71	11.93	11.96	12.12	12.87
Methyl acetate	1.28	1.11	1.07	1.11	1.05	0.85	0.90	0.89	0.89	0.89
Butanal	0.05	0.04	0.04	0.04	0.03	0.03	0.03	0.03	0.02	0.02
Ethyl acetate	1.11	1.09	1.06	1.09	1.03	1.01	1.03	1.02	1.01	1.01
Butan-2-one	0.29	0.28	0.28	0.27	0.27	0.27	0.27	0.27	0.27	0.28
2-methylbutanal	0.15	0.12	0.11	0.11	0.11	0.12	0.12	0.12	0.11	0.11
3-methylbutanal	0.06	0.05	0.05	0.05	0.05	0.05	0.05	0.05	0.05	0.11
Ethanol	12.17	7.85	7.36	7.91	6.62	5.82	5.52	5.67	5.79	5.96
Ethyl propanoate	0.32	0.29	0.29	0.28	0.29	0.28	0.27	0.26	0.26	0.26
3-pentanone	2.41	1.96	1.98	1.97	1.98	2.00	2.02	2.05	2.12	2.20
Butan-2-ol	0.09	0.08	0.08	0.08	0.08	0.05	0.04	0.05	0.05	0.05
Hexanal	2.08	2.71	2.68	2.70	2.74	2.71	2.85	3.04	3.25	3.75
2-methylpropan-1-ol	0.04	0.01	0.02	0.01	0.01	0.01	0.01	0.01	0.01	0.01
1-penten-3-ol	0.44	0.35	0.34	0.35	0.33	0.33	0.31	0.31	0.31	0.31
(E)-2-pentenal	0.16	0.12	0.13	0.14	0.13	0.13	0.14	0.15	0.15	0.16
Butan-1-ol	0.01	0.01	0.01	0.01	0.01	0.01	0.01	0.01	0.01	0.01
Heptanal	0.06	0.24	0.28	0.29	0.30	0.37	0.38	0.38	0.41	0.47
2-methylbutan-1-ol	nd	nd	0.01	0.01	0.01	nd	nd	nd	nd	nd
3-methylbutan-1-ol	0.04	0.04	0.04	0.04	0.04	0.03	0.04	0.04	0.04	0.04
(E)-2-hexenal	5.03	4.53	4.37	4.43	3.97	3.46	3.78	3.78	3.84	3.87
Octan-3-one	0.04	0.03	0.03	0.03	0.03	0.03	0.02	0.02	0.01	0.01
Pentanol	0.01	0.01	0.01	0.01	0.01	0.01	0.01	0.01	0.01	0.06
1-octen-3-one	0.16	0.47	0.37	0.39	0.36	0.37	0.38	0.40	0.41	0.43
Hexyl acetate	0.85	0.76	0.77	0.79	0.79	0.77	0.77	0.76	0.76	0.75
Octan-2-one	0.02	0.02	0.03	0.01	0.01	0.01	0.01	0.01	0.01	0.01
Octanal	0.42	0.64	0.37	0.49	0.48	0.41	0.40	0.42	0.61	0.98
(Z)-3-hexenyl acetate	0.56	0.51	0.46	0.49	0.48	0.48	0.49	0.50	0.51	0.50
(E)-2-heptenal	0.02	0.04	0.06	0.06	0.09	0.07	0.09	0.09	0.10	0.10
6-methyl-5-hepten-2-one	0.01	0.01	0.01	0.01	0.01	0.01	0.01	0.01	0.01	0.02
Hexanol	1.57	1.47	1.49	1.45	1.44	1.48	1.44	1.43	1.41	1.41
(E)-3-hexen-1-ol	0.10	0.10	0.09	0.10	0.09	0.11	0.11	0.11	0.11	0.11
(Z)-3-hexen-1-ol	0.16	0.14	0.13	0.14	0.14	0.12	0.13	0.13	0.12	0.12
Nonanal	0.19	0.58	0.60	0.62	0.64	0.70	0.69	0.67	0.72	0.80
1-octen-3-ol	0.05	0.07	0.08	0.07	0.07	0.09	0.09	0.09	0.09	0.08
(E)-2-hexen-1-ol	0.50	0.43	0.38	0.39	0.39	0.35	0.38	0.38	0.37	0.37
(Z)-2-hexen-1-ol	0.29	0.30	0.28	0.28	0.27	0.27	0.28	0.28	0.29	0.30
Acetic acid	5.16	7.24	7.63	7.63	7.59	7.08	5.99	5.92	6.25	6.36
Propanoic acid	0.18	0.10	0.13	0.13	0.11	0.15	0.16	0.16	0.18	0.19
Butanoic acid	0.13	0.12	0.11	0.11	0.11	0.10	0.09	0.08	0.07	0.06
2-methylpropanoic acid	0.07	0.06	0.06	0.05	0.05	0.05	0.05	0.05	0.05	0.05
(E)-2-decenal	0.12	0.51	0.74	0.75	0.77	0.75	0.82	0.79	0.80	0.80
Pentanoic acid	0.13	0.14	0.12	0.11	0.13	0.14	0.16	0.19	0.23	0.24
Hexanoic acid	0.57	0.59	0.62	0.62	0.71	0.78	0.77	0.81	1.07	1.22
Heptanoic acid	3.99	3.69	3.93	2.88	2.04	1.84	1.71	1.65	1.62	1.52
Octanoic acid	1.77	1.74	1.55	1.70	1.79	2.12	1.93	1.74	1.66	1.57
Nonanoic acid	0.02	0.03	0.05	0.05	0.06	0.07	0.08	0.07	0.07	0.05

Note: nd, not detected.

Table S3. Concentration of volatile compounds (mg/kg) in VOO3 during the storage experiment.

**Months of storage**	**0**	**3**	**6**	**9**	**12**	**15**	**18**	**21**	**24**	**27**
Octane	0.65	1.51	3.93	8.76	11.59	12.34	13.47	14.06	14.00	13.95
Methyl acetate	0.13	0.16	0.16	0.16	0.16	0.16	0.17	0.17	0.18	0.18
Butanal	0.07	0.06	0.05	0.05	0.04	0.04	0.03	0.03	0.03	0.03
Ethyl acetate	0.16	0.18	0.20	0.19	0.20	0.20	0.20	0.20	0.20	0.19
Butan-2-one	0.55	0.50	0.49	0.48	0.47	0.41	0.40	0.38	0.42	0.41
2-methylbutanal	0.05	0.06	0.07	0.07	0.07	0.08	0.07	0.07	0.08	0.08
3-methylbutanal	0.04	0.05	0.06	0.07	0.07	0.07	0.08	0.08	0.08	0.08
Ethanol	21.09	19.74	21.02	20.95	17.93	15.00	15.19	15.27	15.43	15.67
Ethyl propanoate	0.24	0.20	0.21	0.20	0.23	0.27	0.30	0.29	0.29	0.29
3-pentanone	3.46	3.54	3.35	3.09	2.89	2.80	2.76	2.76	2.79	2.82
Butan-2-ol	0.09	0.07	0.05	0.05	0.05	0.04	0.03	0.04	0.04	0.04
Hexanal	2.62	2.39	2.39	2.30	2.27	2.24	2.26	2.19	2.19	2.19
2-methylpropan-1-ol	0.02	0.01	0.01	0.01	0.01	0.01	0.01	0.01	0.01	0.01
1-penten-3-ol	0.51	0.58	0.58	0.56	0.56	0.56	0.56	0.55	0.54	0.52
(E)-2-pentenal	0.26	0.31	0.29	0.30	0.28	0.26	0.27	0.28	0.30	0.30
Butan-1-ol	0.10	0.02	0.01	0.01	0.03	0.01	0.01	0.01	0.01	0.01
Heptanal	0.05	0.07	0.13	0.20	0.21	0.26	0.26	0.27	0.27	0.30
2-methylbutan-1-ol	0.01	0.01	0.01	0.02	0.02	0.03	0.03	0.03	0.04	0.05
3-methylbutan-1-ol	0.02	0.02	0.02	0.01	0.01	0.01	0.01	0.01	0.01	0.01
(E)-2-hexenal	5.81	3.84	3.30	3.22	3.21	3.18	3.19	3.24	3.13	3.31
Octan-3-one	0.15	0.09	0.09	0.08	0.08	0.08	0.09	0.10	0.09	0.09
Pentanol	0.06	0.03	0.03	0.03	0.03	0.03	0.03	0.03	0.03	0.03
1-octen-3-one	0.11	0.11	0.10	0.11	0.10	0.10	0.11	0.11	0.11	0.11
Hexyl acetate	1.69	1.70	1.65	1.62	1.60	1.54	1.54	1.52	1.51	1.52
Octan-2-one	0.12	0.03	0.03	0.03	0.03	0.03	0.04	0.04	0.04	0.04
Octanal	1.01	1.02	0.82	0.82	0.82	0.77	0.84	0.90	0.93	1.00
(Z)-3-hexenyl acetate	1.73	1.01	0.89	0.89	0.82	0.92	0.99	0.99	0.98	0.98
(E)-2-heptenal	0.02	0.01	0.02	0.05	0.05	0.06	0.06	0.08	0.08	0.08
6-methyl-5-hepten-2-one	0.02	0.01	0.01	0.01	0.01	0.01	0.01	0.01	0.01	0.02
Hexanol	1.65	1.70	1.66	1.66	1.61	1.49	1.48	1.43	1.43	1.43
(E)-3-hexen-1-ol	0.21	0.14	0.11	0.11	0.10	0.09	0.09	0.09	0.09	0.09
(Z)-3-hexen-1-ol	0.29	0.18	0.16	0.16	0.14	0.15	0.16	0.16	0.16	0.16
Nonanal	0.24	0.21	0.30	0.34	0.39	0.37	0.37	0.40	0.40	0.39
1-octen-3-ol	0.14	0.09	0.09	0.08	0.07	0.06	0.06	0.05	0.04	0.04
(E)-2-hexen-1-ol	0.85	0.53	0.46	0.43	0.41	0.42	0.47	0.46	0.45	0.44
(Z)-2-hexen-1-ol	0.59	0.41	0.31	0.31	0.29	0.24	0.24	0.24	0.24	0.23
Acetic acid	2.94	2.73	3.38	3.56	3.77	3.70	3.68	3.76	3.78	3.78
Propanoic acid	0.13	0.13	0.14	0.15	0.16	0.16	0.18	0.19	0.19	0.20
Butanoic acid	0.25	0.34	0.36	0.32	0.29	0.28	0.25	0.25	0.25	0.24
2-methylpropanoic acid	0.10	0.08	0.07	0.07	0.06	0.06	0.05	0.06	0.06	0.06
(E)-2-decenal	2.75	2.53	2.55	2.67	2.59	3.09	3.38	3.37	3.37	3.37
Pentanoic acid	0.29	0.18	0.19	0.17	0.14	0.14	0.14	0.15	0.15	0.15
Hexanoic acid	2.51	1.70	1.33	1.28	0.80	0.66	0.59	0.60	0.58	0.57
Heptanoic acid	4.96	3.89	2.75	3.03	2.25	1.73	1.79	1.30	1.25	1.16
Octanoic acid	4.47	2.93	2.53	2.22	2.02	1.57	1.42	1.26	1.33	1.33
Nonanoic acid	0.12	0.10	0.08	0.08	0.05	0.02	0.02	0.02	0.02	0.02

Table S4. Concentration of volatile compounds (mg/kg) in VOO4 during the storage experiment.

Months of storage	0	3	6	9	12	15	18	21	24	27
Octane	2.01	9.73	10.47	10.84	10.92	11.50	11.51	12.49	13.50	14.13
Methyl acetate	1.03	0.92	0.86	0.94	0.81	0.74	0.66	0.61	0.55	0.49
Butanal	0.04	0.04	0.04	0.04	0.04	0.03	0.03	0.03	0.03	0.03
Ethyl acetate	1.59	1.34	1.30	1.28	1.24	1.17	1.20	1.09	1.05	1.00
Butan-2-one	0.12	0.13	0.14	0.13	0.11	0.11	0.11	0.10	0.10	0.10
2-methylbutanal	0.16	0.13	0.13	0.12	0.12	0.13	0.14	0.12	0.12	0.12
3-methylbutanal	0.09	0.08	0.08	0.07	0.07	0.09	0.09	0.08	0.07	0.05
Ethanol	15.88	16.12	15.78	14.84	12.95	12.07	12.05	10.93	10.10	9.45
Ethyl propanoate	0.10	0.10	0.09	0.08	0.08	0.07	0.07	0.06	0.06	0.05
3-pentanone	2.23	2.17	2.16	2.05	1.95	1.87	1.90	1.68	1.67	1.60
Butan-2-ol	0.06	0.06	0.05	0.05	0.05	0.03	0.03	0.03	0.03	0.02
Hexanal	2.41	2.46	2.38	2.41	2.50	2.70	2.75	2.73	2.72	2.72
2-methylpropan-1-ol	0.01	0.01	0.01	0.01	0.01	0.02	0.02	0.02	0.02	0.02
1-penten-3-ol	0.40	0.37	0.37	0.37	0.35	0.34	0.34	0.32	0.32	0.31
(E)-2-pentenal	0.12	0.11	0.13	0.14	0.18	0.24	0.26	0.26	0.29	0.31
Butan-1-ol	0.04	0.04	0.04	0.04	0.04	0.03	0.03	0.03	0.03	0.03
Heptanal	0.08	0.26	0.29	0.32	0.30	0.35	0.38	0.39	0.39	0.42
2-methylbutan-1-ol	0.02	0.03	0.03	0.03	0.03	0.03	0.03	0.03	0.03	0.03
3-methylbutan-1-ol	0.08	0.05	0.05	0.05	0.05	0.06	0.06	0.06	0.06	0.06
(E)-2-hexenal	5.71	4.89	4.90	4.92	4.48	3.76	3.47	3.36	2.99	2.57
Octan-3-one	0.02	0.03	0.03	0.03	0.03	0.06	0.07	0.08	0.08	0.08
Pentanol	0.01	nd	nd	nd	0.01	0.01	0.01	0.01	0.01	0.01
1-octen-3-one	0.10	0.15	0.15	0.15	0.15	0.17	0.17	0.18	0.19	0.20
Hexyl acetate	0.89	0.81	0.81	0.79	0.78	0.77	0.75	0.72	0.72	0.70
Octan-2-one	0.02	0.01	0.01	0.01	0.01	nd	0.01	0.01	0.01	0.01
Octanal	0.55	0.45	0.41	0.47	0.45	0.40	0.38	0.34	0.34	0.31
(Z)-3-hexenyl acetate	0.48	0.40	0.40	0.40	0.38	0.36	0.36	0.35	0.34	0.31
(E)-2-heptenal	0.02	0.05	0.06	0.06	0.07	0.06	0.07	0.08	0.08	0.09
6-methyl-5-hepten-2-one	0.01	0.01	0.01	0.01	0.01	0.01	0.01	0.01	nd	0.01
Hexanol	1.08	0.97	0.97	0.96	0.93	0.95	0.94	0.90	0.89	0.88
(E)-3-hexen-1-ol	0.05	0.04	0.04	0.04	0.04	0.04	0.04	0.04	0.03	0.03
(Z)-3-hexen-1-ol	0.05	0.04	0.04	0.04	0.04	0.04	0.04	0.04	0.04	0.04
Nonanal	0.22	0.56	0.61	0.69	0.69	0.63	0.60	0.60	0.60	0.61
1-octen-3-ol	0.05	0.06	0.06	0.06	0.07	0.07	0.08	0.08	0.08	0.09
(E)-2-hexen-1-ol	0.25	0.22	0.21	0.21	0.19	0.19	0.19	0.20	0.19	0.19
(Z)-2-hexen-1-ol	0.16	0.14	0.14	0.14	0.14	0.14	0.14	0.13	0.13	0.13
Acetic acid	0.63	1.19	1.18	1.09	1.03	0.92	0.86	0.83	0.82	0.79
Propanoic acid	0.10	0.13	0.11	0.09	0.10	0.11	0.11	0.13	0.13	0.13
Butanoic acid	0.25	0.24	0.23	0.22	0.21	0.18	0.17	0.17	0.16	0.15
2-methylpropanoic acid	0.06	0.06	0.05	0.05	0.05	0.05	0.05	0.05	0.05	0.04
(E)-2-decenal	2.55	2.66	2.95	2.88	2.99	3.13	3.39	3.47	3.46	3.47
Pentanoic acid	0.11	0.12	0.12	0.12	0.14	0.16	0.17	0.20	0.20	0.20
Hexanoic acid	0.63	0.85	0.85	0.92	1.05	1.01	1.04	1.08	1.18	1.23
Heptanoic acid	1.15	1.16	1.38	1.57	2.82	3.23	3.27	3.31	3.67	4.00
Octanoic acid	1.03	1.31	1.14	1.40	1.46	1.74	1.82	1.89	2.06	2.14
Nonanoic acid	0.02	0.02	0.02	0.02	0.03	0.04	0.04	0.05	0.05	0.05

Note: nd, not detected.

Table S5. Concentration of the chemical series of the volatile compounds identified in the oils (mg/kg) at two different moments (before and after the storage experiment).

**Months of storage**	**VOO1**		**VOO2**		**VOO3**		**VOO4**	
	**0**	**27**	**0**	**27**	**0**	**27**	**0**	**27**
Aldehydes	9.47	5.74	8.34	11.11	12.91	11.12	11.96	10.70
Alcohols	28.25	21.08	15.46	8.78	25.63	18.70	18.13	11.27
Esters	4.46	4.11	4.13	3.42	3.95	3.18	4.07	2.56
Ketones	5.65	4.53	2.93	2.94	4.39	3.45	2.50	1.98
Carboxylic acids	6.41	9.75	12.02	11.25	15.75	7.51	3.97	8.73
